# Supplementary material for: Developing an evaluation approach for the in-depth review of a new undergraduate medical programme as a complex system
Source: PLoS One. 2024 Dec 31;19(12):e0312730. doi: 10.1371/journal.pone.0312730 (PMC11687765; doi:10.1371/journal.pone.0312730)
Supplement: S1 Text — (DOCX) [file pone.0312730.s001.docx]

| **MBBS Review Steering Committee 2015**  Faculty of Medicine, University of Botswana | 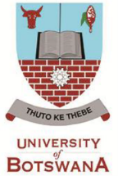 |
| --- | --- |

**WFME standards: summary**

NOTE: only the ‘Basic’ standards were used for the Review

| *Area and sub-area* | *Standard – basic (B)* |
| --- | --- |
| **1. MISSION AND OUTCOMES** | |
| **1.1 STATEMENT OF MISSION** | The medical school **must**   - define its mission and make it known to its constituency and the health sector it serves. (B 1.1.1) - in its mission statement outline the aims and the educational strategy resulting in a medical doctor - competent at a basic level. (B 1.1.2) - with an appropriate foundation for future career in any branch of medicine. (B 1.1.3) - capable of undertaking the roles of doctors as defined by the health sector. (B 1.1.4) - prepared and ready for postgraduate medical training (B 1.1.5) - committed to lifelong learning (B 1.1.6) - ensure that the mission encompasses the health needs of the community, the needs of the health care system and other aspects of social accountability. (B 1.1.7) |
| **1.2 PARTICIPATION IN FORMULATION OF MISSION** | The medical school **must**   - ensure that its principal stakeholders participate in formulating the mission. (B 1.2.1) |
| **1.3 INSTITUTIONAL AUTONOMY AND ACADEMIC FREEDOM** | The medical school **must** have institutional  autonomy to   - formulate and implement policies for which its faculty/academic staff and administration are responsible, especially regarding - design of the curriculum. (B 1.3.1) - use of the allocated resources necessary for implementation of the curriculum. (B 1.3.2) |
| **1.4 EDUCATIONAL OUTCOMES** | The medical school **must**   - define the intended educational outcomes that students should exhibit upon graduation in relation to - their achievements at a basic level regarding knowledge, skills, and attitudes (B 1.4.1) - appropriate foundation for future career in any branch of medicine (B 1.4.2) - their future roles in the health sector. (B 1.4.3) - their subsequent postgraduate training (B 1.4.4) - their commitment to and skills in lifelong learning. (B 1.4.5) - the health needs of the community, the needs of the health care system and other aspects of social accountability. (B 1.4.6) - ensure appropriate student conduct with respect to fellow students, faculty members, other health care personnel, patients and their relatives. (B 1.4.7) |
| **2. EDUCATIONAL PROGRAMME** | |
| **2.1 CURRICULUM MODEL AND**  **INSTRUCTIONAL METHODS** | The medical school **must**   - define the curriculum model. (B 2.1.1) - define the instructional and learning methods employed. (B 2.1.2) - ensure that the curriculum prepares the students for lifelong learning. (B 2.1.3) - ensure that the curriculum is delivered in accordance with principles of equality. (B 2.1.4) |
| **2.2 SCIENTIFIC METHOD** | The medical school **must**   - throughout the curriculum teach - the principles of scientific method, including analytical and critical thinking. (B 2.2.1) - medical research methods. (B 2.2.2) - evidence-based medicine. (B 2.2.3) |
| **2.3 BASIC BIOMEDICAL SCIENCES** | The medical school **must**   - in the curriculum identify and incorporate - the contributions of the basic biomedical sciences to create understanding of scientific knowledge. (B 2.3.1) - concepts and methods fundamental to acquiring and applying clinical science. (B 2.3.2) |
| **2.4 BEHAVIOURAL AND SOCIAL SCIENCES AND MEDICAL ETHICS** | The medical school **must**   - in the curriculum identify and incorporate the contributions of the: - behavioural sciences. (B 2.4.1) - social sciences. (B 2.4.2) - medical ethics. (B 2.4.3) - medical jurisprudence. (B 2.4.4) |
| **2.5 CLINICAL SCIENCES AND SKILLS** | The medical school **must**   - in the curriculum identify and incorporate the contributions of the clinical sciences to ensure that students - acquire sufficient knowledge and clinical and professional skills to assume appropriate responsibility after graduation. (B 2.5.1) - spend a reasonable part of the programme in planned contact with patients in relevant clinical settings. (B 2.5.2) - experience health promotion and preventive medicine (B 2.5.3) - specify the amount of time spent in training in major clinical disciplines. (B 2.5.4) - organise clinical training with appropriate attention to patient safety. (B 2.5.5) |
| **2.6 CURRICULUM STRUCTURE,**  **COMPOSITION AND DURATION** | The medical school **must**   - describe the content, extent and sequencing of courses and other curricular elements to ensure appropriate coordination between basic biomedical, behavioural and social and clinical subjects. (B 2.6.1) |
| **2.7 PROGRAMME MANAGEMENT** | The medical school **must**   - have a curriculum committee, which under the governance of the academic leadership (the dean) has the responsibility and authority for planning and implementing the curriculum to secure its intended educational outcomes. (B 2.7.1) - in its curriculum committee ensure representation of staff and students. (B 2.7.2) |
| **2.8 LINKAGE WITH MEDICAL PRACTICE AND THE HEALTH**  **SECTOR** | The medical school **must**   - ensure operational linkage between the educational programme and the subsequent stages of training or practice after graduation. (B 2.8.1) |
| **3. ASSESSMENT OF STUDENTS** | |
| **3.1 ASSESSMENT METHODS** | The medical school **must**   - define, state and publish the principles, methods and practices used for assessment of its students, including the criteria for setting pass marks, grade boundaries and number of allowed retakes. (B 3.1.1) - ensure that assessments cover knowledge, skills and attitudes (B 3.1.2) - use a wide range of assessment methods and formats according to their “assessment utility” (B 3.1.3) - ensure that methods and results of assessments avoid conflicts of interest (B 3.1.4) - ensure that assessments are open to scrutiny by external expertise. (B 3.1.5) |
| **3.2 RELATION BETWEEN**  **ASSESSMENT AND LEARNING** | The medical school **must**   - use assessment principles, methods and practices that - are clearly compatible with intended educational outcomes and instructional methods. (B 3.2.1) - ensure that the intended educational outcomes are met by the students. (B 3.2.2) - promote student learning. (B 3.2.3) - provide an appropriate balance of formative and summative assessment to guide both learning and decisions about academic progress. (B 3.2.4) |
| **4. STUDENTS** | |
| **4.1 ADMISSION POLICY AND**  **SELECTION** | The medical school **must**   - formulate and implement an admission policy based on principles of objectivity, including a clear statement on the process of selection of students. (B 4.1.1) - have a policy and implement a practice for admission of disabled students ( B 4.1.2) - have a policy and implement a practice for transfer of students from other programmes and institutions (B 4.1.3) |
| **4.2 STUDENT INTAKE** | The medical school **must**   - define the size of student intake and relate it to its capacity at all stages of the programme. (B 4.2.1) |
| **4.3 STUDENT COUNSELLING AND**  **SUPPORT** | The medical school and/or the University **must**   - have a system for academic counselling of its student population. (B 4.3.1) - offer a programme of student support, addressing social, financial and personal needs. (B 4.3.2) - allocate resources for student support. (B 4.3.3) - ensure confidentiality in relation to counselling and support. (B 4.3.4) |
| **4.4 STUDENT REPRESENTATION** | The medical school **must**   - formulate and implement a policy, that ensures participation of student representatives and appropriate participation in the design, management and evaluation of the curriculum, and in other matters relevant to students. (B 4.4.1) |
| **5. ACADEMIC STAFF/FACULTY** | |
| **5.1 RECRUITMENT AND SELECTION POLICY** | The medical school **must**   - formulate and implement a staff recruitment and selection policy which - outlines the type, responsibilities and balance of the academic staff/faculty of the basic biomedical sciences, the behavioural and social sciences and the clinical sciences required to deliver the curriculum adequately, including the balance between medical and non-medical academic staff, the balance between full-time and part-time academic staff, and the balance between academic and non-academic staff. (B 5.1.1) - addresses criteria for scientific, educational and clinical merit, including the balance between teaching, research and service qualifications. (B 5.1.2) - specifies and monitors the responsibilities of its academic staff/faculty of the basic biomedical sciences, the behavioural and social sciences and the clinical sciences.(B 5.1.3) |
| **5.2 STAFF ACTIVITY AND DEVELOPMENT POLICY** | The medical school **must**   - formulate and implement a staff activity and development policy which - allows a balance of capacity between teaching, research and service functions. (B 5.2.1) - ensures recognition of meritorious academic activities, with appropriate emphasis on teaching, research and service qualifications. (B 5.2.2) - ensures that clinical service functions and research are used in teaching and learning. (B 5.2.3) - ensures sufficient knowledge by individual staff members of the total curriculum. (B 5.2.4) - includes teacher training, development, support and appraisal. (B 5.2.5) |
| **6. EDUCATIONAL RESOURCES** | |
| **6.1 PHYSICAL FACILITIES** | The medical school **must**   - have sufficient physical facilities for staff and students to ensure that the curriculum can be delivered adequately. (B 6.1.1) - ensure a learning environment, which is safe for staff, students, patients and their carers. (B 6.1.2) |
| **6.2 CLINICAL TRAINING RESOURCES** | The medical school **must**   - ensure necessary resources for giving the students adequate clinical experience, including sufficient - number and categories of patients. (B 6.2.1) - clinical training facilities. (B 6.2.2) - supervision of their clinical practice. (B 6.2.3) |
| **6.3 INFORMATION TECHNOLOGY** | The medical school **must**   - formulate and implement a policy which addresses effective use and evaluation of appropriate information and communication technology in the educational programme. (B 6.3.1) |
| **6.4 MEDICAL RESEARCH AND**  **SCHOLARSHIP** | The medical school **must**   - use medical research and scholarship as a basis for the educational curriculum. (B 6.4.1) - formulate and implement a policy that fosters the relationship between medical research and education. (B 6.4.2) - describe the research facilities and priorities at the institution. (B 6.4.3) |
| **6.5 EDUCATIONAL EXPERTISE** | The medical school **must**   - have access to educational expertise where required. (B 6.5.1) - formulate and implement a policy on the use of educational expertise - in curriculum development.(B 6.5.2) - in development of teaching and assessment methods. (B 6.5.3) |
| **6.6 EDUCATIONAL EXCHANGES** | The medical school **must**   - formulate and implement a policy for - national and international collaboration with other educational institutions. (B 6.6.1) - transfer of educational credits. (B 6.6.2) |
| **7. PROGRAMME EVALUATION** | |
| **7.1 MECHANISMS FOR PROGRAMME MONITORING AND EVALUATION** | The medical school **must**   - have a programme of routine curriculum monitoring of processes and outcomes. (B 7.1.1) - establish and apply a mechanism for programme evaluation that - addresses the curriculum and its main components. (B 7.1.2) - addresses student progress. (B 7.1.3) - identifies and addresses concerns.(B 7.1.4) - ensure that relevant results of evaluation influence the curriculum. (B 7.1.5) |
| **7.2 TEACHER AND STUDENT FEEDBACK** | The medical school **must**   - systematically seek, analyse and respond to teacher and student feedback. (B 7.2.1) |
| **7.3 PERFORMANCE OF STUDENTS AND GRADUATES** | The medical school **must**   - analyse performance of cohorts of students and graduates in relation to its - mission and intended educational outcomes. (B 7.3.1) - curriculum. (B 7.3.2) - provision of resources. (B 7.3.3) |
| **7.4 INVOLVEMENT OF STAKEHOLDERS** | The medical school **must**   - in its programme monitoring and evaluation activities involve - its academic staff and students. (B 7.4.1) - its governance and management.(B 7.4.2) |
| **8. GOVERNANCE AND ADMINISTRATION** | |
| **8.1 GOVERNANCE** | The medical school **must**   - define its governance structures and functions including their relationships within the University. (B 8.1.1) |
| **8.2 ACADEMIC LEADERSHIP** | The medical school **must**   - describe the responsibilities of its academic leadership for definition and management of the medical educational programme. (B 8.2.1) |
| **8.3 EDUCATIONAL BUDGET AND**  **RESOURCE ALLOCATION** | The medical school **must**   - have a clear line of responsibility and authority for resourcing the curriculum, including a dedicated educational budget. (B 8.3.1) - allocate the resources necessary for the implementation of the curriculum and distribute the educational resources in relation to educational needs. (B 8.3.2) |
| **8.4 ADMINISTRATIVE STAFF AND**  **MANAGEMENT** | The medical school **must**   - have an administrative and professional staff that is appropriate to - support implementation of its educational programme and related activities. (B 8.4.1) - ensure good management and resource deployment. (B 8.4.2) |
| **8.5 INTERACTION WITH HEALTH**  **SECTOR** | The medical school **must**   - have constructive interaction with the health and health related sectors of society and government. (B 8.5.1) |
| **9. CONTINUOUS RENEWAL** | |
|  | **Basic standard:**  The medical school **must** as a dynamic and socially accountable institution   - initiate procedures for regularly reviewing and updating its structure and functions. (B 9.0.1) - rectify documented deficiencies. (B 9.0.2) - allocate resources for continuous renewal. (B 9.0.3) |
