# Supplementary material for: Developing an evaluation approach for the in-depth review of a new undergraduate medical programme as a complex system
Source: PLoS One. 2024 Dec 31;19(12):e0312730. doi: 10.1371/journal.pone.0312730 (PMC11687765; doi:10.1371/journal.pone.0312730)
Supplement: S2 Text — (DOCX) [file pone.0312730.s002.docx]

| **MBBS Review Steering Committee 2015**  Faculty of Medicine, University of Botswana | 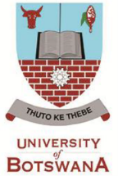 |
| --- | --- |

**5. Standards from UB, FoM, MoH, Global Consensus, Lancet Commission**

1. **Vision, Mission, Values of the University of Botswana (VMVU)**

Vision

The University of Botswana is to be a leading academic centre of excellence in Africa and the world

Mission

**The Mission of the University of Botswana is to improve economic and social conditions for the Nation** (Note 1) **while advancing itself as a distinctively African university with a regional and international outlook. Specifically, the University will:**

- Provide excellence in the delivery of learning to ensure society is provided with talented, creative and confident graduates (Note 2)
- Advance knowledge and understanding through excellence in research and its application
- Improve economic and social development by high impact engagement with business, the professions, government and civil society

The University will fulfil its Vision and Mission by:

- **Offering** quality academic and professional programmes that ensure a commitment to and a mastery of life-long learning skills as well as encouraging a spirit of critical enquiry (Note 3)
- **Developing** a student-centred, intellectually stimulating and technologically advanced teaching, learning and research environment (Note 4)
- **Producing** graduates who are independent, confident, self-directed, critical thinkers, professionally competent, reflective practitioners, innovative, socially responsible and thereby marketable and competitive nationally and internationally (Note 5)
- **Advancing** scholarship and generating research through the discovery, integration, dissemination and application of knowledge (Note 6)
- **Serving** as an intellectual and cultural centre that draws upon the nation's indigenous knowledge base and which promotes Botswana's social and cultural heritage as well as being a community resource for new ideas, partnerships, and collaborative effort (Note 7)
- **Providing** leadership in responding to the nation's cultural, economic, political scientific, social, technological and industrial needs and contributing to the qualitative development of Botswana's higher education system (Note 8)
- **Extending** access to higher education through the utilisation of information and communication technologies, within the framework of life-long and open learning (Note 9)
- **Recruiting** and developing quality staff and students, recognising and valuing the essential contribution they make, as well as rewarding excellence in the work they perform (Note 10)
- **Promoting** the health, social, and spiritual welfare of the University community through a range of policies and programmes and a diversity of positive co-curricular activities and experiences (Note 11)
- **Enhancing** the teaching, learning and research environment through the provision of a proactive style of leadership and management and efficient, effective and quality driven institutional support services. (Note 12)

Values

To achieve its Vision and to fulfil its Mission, the University of Botswana values the following:

- **Academic Excellence**, by creating a holistic environment which ensures that learning is their central focus and by establishing and developing a range of learning, social, cultural and recreational opportunities that will facilitate the full realisation of their potential for academic and personal growth (Note 13)
- **Academic Freedom**, by upholding the spirit of free and critical thought and enquiry, through the tolerance of a diversity of beliefs and understanding, as well as the open exchange of ideas and knowledge (Note 14)
- **Academic Integrity** expressed in creativity, objective analysis, experimentation, critical appraisal, independent thought, informed debate and intellectual honesty (Note 15)
- **Cultural Authenticity** by ensuring that the diversity of Botswana's indigenous values and cultural heritage forms an important part of the academic and organisational life of the institution (Note 16)
- **Internationalism** through participation in the global world of scholarship, by being receptive and responsive to issues within the international environment as well as the recruitment of an international staff and student body (Note 17)
- **Social responsibility** by promoting an awareness of, and providing leadership in responding to, the issues and problems facing society (Note 18)
- **Equity** by ensuring equal opportunity and non-discrimination on the basis of personal, ethnic, religious, gender or other social characteristics (Note 19)
- **Autonomy** as an institution, that is, through its self-governing structures, independent in action while being responsive to societal needs (Note 20)
- **Public Accountability** by ensuring transparent decision-making and open review as well as the full participation of stakeholders in the development of the institution (Note 21)
- **Productivity** through the setting and rewarding of high standards of performance underpinned by a dedication to quality, efficiency and effectiveness throughout the institution.

1. **Vision, Mission, Values of the Faculty of Medicine (VMVF)**

Vision

Our vision is to educate, recruit, develop and retain medical doctors for the national healthcare system (Note 1) and to enhance the health of the people of Botswana and beyond (Note 2).

Mission

The mission of the School of Medicine of the University of Botswana is to prepare skilful, productive, ethical and compassionate physicians who advance and apply in a humanistic and professional manner scientific discovery and technological innovation to the health care needs of individual patients, their families and larger societal groups (Note 3).

Values

The School of Medicine at its inception adopted the Teaching Health System as its preferred method of imparting knowledge and skills to its students (Note 4). This is a System whereby healthcare facilities at all levels are used as teaching and training platforms. This is a marked departure from the traditional way of teaching students where almost all the training was done in academic hospitals.

The School of Medicine utilises referral, district and primary hospitals as well as clinics for clinical teaching, which starts right from the first month of medical training in the 1st Year (Note 5). The curriculum was designed to reflect this in that it is community-based. The benefits of such an approach are twofold:

1. The healthcare facilities stand to benefit from the resources of the School (Note 6), and
2. The School hopes to produce high quality medical practitioners with intimate knowledge and skills to function at all levels of healthcare in the country (Note 7). Research has shown that medical students trained in a particular environment are more likely to stay and work in that environment upon completion of their studies (Note 8). This will help address the brain drain crisis that the Botswana’s health care system is currently facing.

1. **Botswana National Health Policy (2011) (BNHP)**

Relevant sections of the Policy are reproduced below.

**Chapter 2: Situation Analysis**

***2.4 Current Health Status in Botswana***

The life expectancy at birth in Botswana is estimated at 54.4 years (48.8 males: and 60 females). The crude birth and crude death rates were estimated at 29.7 and 11.2 per 1000 respectively while infant and under-five mortality rates were 57 and 76 per 1000 live births respectively. The Maternal Mortality Rate (MMR) is 193 per hundred thousand live births based on the CSO 2007 calculations. A total of 25.9% of the population are stunted, of which 16.8% are moderately stunted and 9.1% severely stunted.

Comment 1: obstetrical and child welfare/ nutritional skills need major attention in the MBBS programme

***2.5 Major Causes of Disease Burden in Botswana***

2.5.1 In Botswana both morbidity and mortality for all ages are still dominated by infectious diseases, with HIV/AIDS and other communicable diseases causing about half of the deaths. Due to an effective ARV programme, mortality due to HIV/AIDS has been declining over the past four years, but still is a major concern.

Comment 2: prevention and management of HIV need major attention in the MBBS programme – so too other common infectious diseases (see below)

2.5.2 The Infant Mortality Rate (IMR) and under-5 mortality rate (U5MR) remain high with year-on-year fluctuations. More than two-third of these deaths is due to communicable diseases, with diarrhoea and pneumonia being the two main killers. More than 40% of infant deaths are within the first week of birth. The Maternal Mortality Rate is also fluctuating. Although non-communicable diseases like hypertension, diabetes, etc. are not among the top ten causes of disease morbidity and mortality, the rates are increasing. Of these, cardiovascular diseases and cancers have been increasing alarmingly over the last decade. Tables 1 and 2 below indicate the major causes of mortality and morbidity in Botswana.

Comment 3: prevention and management of diarrhoeal disease and ARIs and perinatal care need major attention in the MBBS programme

**Table 1: Major causes of Mortality of Public Health Concern – 2006**

| **Diseases/Conditions** | **Numbers** | **Percentage** |
| --- | --- | --- |
| TB  HIV  Other Infectious  Cancers  Anaemias  Endocrine, Metabolic & Nutritional  Cardiovascular diseases  Respiratory Diseases  Digestive System Diseases  Diseases of Nervous System  Injuries/Trauma  All Other Diseases | 731  1,985  2,474  553  401  874  1,233  1,448  400  420  324  603 | 6%  17%  22%  5%  4%  8%  11%  13%  3%  4%  3%  5% |

**Table 2: Major Causes of Inpatient Morbidity (Excluding Neonatal Conditions) All Age Groups – 2006**

| **Diseases and Conditions** | **Number** | **Percentage** |
| --- | --- | --- |
| Intestinal infectious diseases  Tuberculosis  Human immunodeficiency virus (HIV) diseases  Other viral diseases  Pneumonia and other ARI  Pregnancy, Childbirth and the Puerperium  Cancer  Diseases of the Digestive System  Diseases of the Circulatory System  Injury, Poisoning and other external  Total for the above  Other Diseases and conditions | 9,409  4,121  2,965  2,983  8,133  16,415  4,226  5,219  6,298  10,853  70,622  31,730 | 9%  4%  3%  3%  8%  16%  4%  5%  6%  11%  69%  31% |

Comment 4: prevention and management of TB, cardiovascular disease, cancer and trauma need major attention in the MBBS programme

***2.8 Human Resources for Health***

2.8.1 Shortage of trained and qualified staff remains one of the major bottlenecks towards the availability of quality health care in Botswana. There are also increasing demands on the already over-stretched skilled workforce as a result of the addition of further programmes and projects, in particular those related to HIV/AIDS. Although the rate of attrition is negligible, there is a high turnover of staff at all levels of the health sector. Other challenges relate to inequitable deployment and failure to optimise the existing skill mix. An appropriate division of labour could contribute significantly to reducing the apparent shortage of staff.

Comment 5: it is important to train doctors who are able to function at all levels in the health care system and who work well with other members of the health care team

2.8.2 The training of health care professionals is provided for by a combination of in-country and out-of-country institutions, with a heavy reliance on out-of-country arrangements. There are eight training institutes for nurses and some areas of health technologies only. In addition, the University of Botswana produces a limited number of nurses and some health technologists. The country’s first medical school is currently under development. Due to this limited production of skilled health professionals, a large number of expatriates are deployed in the health sector.

**Chapter 3: Vision, Mission and Guiding Principles**

Botswana recognises health as a basic necessity and the need to promote health as imperative for social justice. This is best clarified through the following vision, mission and guiding principles.

Comment 6: practical health promotion must be a key component of the MBBS programme

Comment 7: social justice is a national priority so social accountability must be reflected in the MBBS programme

***3.1 Vision***

An enabling environment in which all people living in Botswana have the opportunity to achieve and maintain the highest level of health and well-being.

***3.2 Mission Statement***

A sustainable improvement in health status through progressive creation and maintenance of physical, mental, economic and social well-being.

Comment 8: the doctors produced by the MBBS programme must play a significant role in uplifting the health and wellbeing of all the people of Botswana

***3.3 Guiding Principles/Values***

The following principles will guide the implementation of the Botswana National Health Policy:

| **Guiding Principle** | **Description** |
| --- | --- |
| ***Ethics*** | Respect for human dignity, rights, confidentiality and cultural beliefs. |
| ***Norms and Standards*** | Good management practices and quality assurance in service delivery.  Comment 9: the doctors produced by the MBBS programme must be good managers |
| ***Equity*** | Equitable distribution of resources to guarantee accessibility to quality services at every point of demand, especially for the vulnerable, marginalised and underserved, irrespective of political, ethnic or religious affiliations and place of domicile.  Comment 10: the MBBS programme must take steps (e.g. in student selection and selection of the sites where training takes place) to promote retention of graduates in underserved areas and populations |
| ***Ownership*** | Involvement/participation of all stakeholders (providers and users) of health services in defining policy as well as the implementation framework.  Comment 11: the FoM must work closely with MoH, NGOs, BHPC and other stakeholders (including the ‘community’, however that is defined) in planning and implementing the MBBS programme |
| ***Evidence-based*** | The policy will be based on evidence particularly pertaining to Botswana.  Comment 12: information about morbidity and mortality in Botswana must guide the content and priorities in the MBBS programme |
| ***Innovation*** | Continuous exploration of new ideas in health care delivery, e.g. geographical targeting to benefit high priority areas; health insurance coverage for the disadvantaged sections of society; public-private partnership; demand-side financing etc.  See Comment 10 |
| ***Gender Equity*** | Addressing gender sensitive and responsive issues, including the equal involvement of men and women in decision-making, eliminating obstacles (barriers) to services utilisation, and the prevention of gender-based violence. |
| ***Client Satisfaction*** | Ensuring efficient twenty-four hour quality health services that is more responsive and sensitive to customer needs. |
| ***Skilled Staff Retention and Circulation*** | Attractive service conditions (package) and job satisfaction to encourage a net inflow of critically required skills. |
| ***Partnerships*** | Increasing community empowerment; active involvement of the private sector, NGOs, local government authorities and civil society, and effective development partner co-ordination.  See Comment 11 |

**Chapter 4: Policy Thrusts / Principal Areas of Action**

***4.3 Human Resources for Health***

Human resources for health (HRH) are the backbone of service delivery in the health sector. Creating an appropriately skilled, highly motivated, client-focused health workforce is critical for Botswana to attain its ambition of ensuring an enabling environment in which all people living in Botswana have the opportunity to reach and maintain the highest attainable level of health.

Comment 13: the MBBS programme must produce skilled, motivated, client-focused doctors

***4.3.1 Goal***

Ensuring an appropriately skilled, motivated, well distributed and productive workforce for the provision of quality health services effectively and efficiently, delivered to all the people living in Botswana.

See Comment 10

***4.3.2 Objectives***

- To strategically plan health workforce development for the sector;
- To develop and continuously review recruitment and retention strategies for the health workforce;
- To strengthen the management of human resources through the development and implementation of performance standards and norms for efficient service delivery.

See Comment 13

***4.3.3 Policy Initiatives***

1. The MOH shall strategically forecast HRH needs, taking into account the multiplicity of professions and skills, service delivery facilities and providers (public, private and NGOs), population health needs and their growth, and geographical distribution.

Comment 14: the FoM must be in constant communication with MoH about the need for doctors

Comment 15: the MBBS admissions policy must have a geographical component

1. The MOH in collaboration with its partners shall develop and review health workforce strategic plans from time-to-time.

See Comment 14

1. The MOH in collaboration with the Ministry of Education and Skills Development shall ensure the equitable production of an adequate and appropriately skilled health workforce to provide health services at all levels of health care delivery.

See Comments 5, 10, 13

1. The MOH in collaboration with its partners shall harmonise the recruitment and deployment criteria of the health workforce to reduce turnover and ensure continuity of care.
2. The MOH in collaboration with the Directorate of Public Service Management (DPSM) and other relevant government sectors shall periodically review the conditions of service (salary, housing, professional advancement, contractual obligations, involvement in decision-making, recognition of staff contribution and other incentives) and develop appropriate recruitment and retention strategies, both for national and expatriate health workers, within the public sector.
3. The MOH shall ensure that all data generated in pre- and in-service training, recruitment, deployment and migration of health workers be captured, stored in a database, analysed and interpreted for decision-making, and to inform future national policy directions.
4. The MOH shall ensure that the IHSP incorporates the Health Workforce Strategic Plan, such that the right number of staff with the right skills is in the right place to deliver the package of services.

See Comment 14

1. The MOH shall develop and periodically update the staff norms/skills-mix by care level based on research which includes users’ views, to ensure well informed pre-service training and efficient recruitment and deployment of the health workforce, and to ensure an uninterrupted provision of essential health services.

See Comments 1-4 and 14

1. The government shall develop a regulatory mechanism for alternate medical (traditional-herbalist, homeopathy, etc.) practices, preferably in the form of an Act to be administered by MOH and the Traditional Practitioners’ Association through the creation of a registration system for alternate medical practitioners, in order to safeguard against malpractice and misconduct.
2. The government shall promote the formation of and strengthen professional associations and unions to ensure well informed involvement in decision-making and the amicable settling of disputes.
3. The MOH shall periodically review and update the Health Professionals Act and the Nurses and Midwifery Act in order to modernise the accreditation process (for instance, incorporating periodic testing for the continuation of professional registration).
4. **Global Consensus for Social Accountability of Medical Schools (GCSA)**

Ten strategic directions of the global consensus

Direction 1: Anticipating society’s health needs

Direction 2: Partnering with the health system and other stakeholders

Direction 3: Adapting to the evolving roles of doctors

Direction 4: Fostering outcome-based education

Direction 5: Responsive and responsible governance of the medical school

Direction 6: Refining the standards for education, research and service delivery

Direction 7: Continuous quality improvement in education, research, service

Direction 8: Establishing mandated mechanisms for accreditation

Direction 9: Balancing global principles with context specificity

Direction 10: Defining the role of society

1. **The Lancet Commission report (LC)**

Proposed reforms

Instructional reforms should encompass the entire range from admission to graduation, to generate a diverse student body with a competency-based curriculum that, through the creative use of information technology (IT), prepares students for the realities of teamwork, to develop flexible career paths that are based on the spirit and duty of a new professionalism.

1. Adoption of competency-based curricula that are responsive to rapidly changing needs rather than being dominated by static coursework. Competencies should be adapted to local contexts and be determined by national stakeholders, while harnessing global knowledge and experiences. Simultaneously, the present gaps should be filled in the range of competencies that are required to deal with 21^st^ century challenges common to all countries—e.g. the response to global health security threats or the management of increasingly complex health systems.
2. Promotion of interprofessional and transprofessional education that breaks down professional silos while enhancing collaborative and non-hierarchical relationships in effective teams. Alongside specific technical skills, interprofessional education should focus on cross-cutting generic competencies, such as analytical abilities (for effective use of both evidence and ethical deliberation in decision making), leadership and management capabilities (for efficient handling of scarce resources in conditions of uncertainty), and communication skills (for mobilisation of all stakeholders, including patients and populations).
3. Exploitation of the power of IT for learning through development of evidence, capacity for data collection and analysis, simulation and testing, distance learning, collaborative connectivity, and management of the increase in knowledge. Universities and similar institutions have to make the necessary adjustments to harness the new forms of transformative learning made possible by the IT revolution, moving beyond the traditional task of transmitting information to the more challenging role of developing the competencies to access, discriminate, analyse, and use knowledge. More than ever, these institutions have the duty of teaching students how to think creatively to master large flows of information in the search for solutions.
4. Adaptation locally but harnessing of resources globally in a way that confers capacity to flexibly address local challenges while using global knowledge, experience, and shared resources, including faculty, curriculum, didactic materials, and students linked internationally through exchange programmes.
5. Strengthening of educational resources, since faculty, syllabuses, didactic materials, and infrastructure are necessary instruments to achieve competencies. Many countries have severe deficits that require mobilising resources, both financial and didactic, including open access to journals and teaching materials. Faculty development needs special attention through increased investments in education of educators, stable and rewarding career paths, and constructive assessment linked to incentives for good performance.
6. Promote a new professionalism that uses competencies as the objective criterion for the classification of health professionals, transforming present conventional silos. A set of common attitudes, values, and behaviours should be developed as the foundation for preparation of a new generation of professionals to complement their learning of specialties of expertise with their roles as accountable change agents, competent managers of resources, and promoters of evidence-based policies. Institutional reforms should align national eff orts through joint planning especially in the education and health sectors, engage all stakeholders in the reform process, extend academic learning sites into communities, develop global collaborative networks for mutual strengthening, and lead in promotion of the culture of critical inquiry and public reasoning.
7. Establishment of joint planning mechanisms in every country to engage key stakeholders, especially ministries of education and health, professional associations, and the academic community, to overcome fragmentation by assessment of national conditions, setting priorities, shaping policies, tracking change, and harmonising the supply of and demand for health professionals to meet the health needs of the population. In this planning process, special attention should be paid to sex and geography. As the proportion of women in the health workforce increases, equal opportunities need to be in place—e.g. through more flexible working arrangements, career paths that accommodate temporary breaks, support to other social roles of women such as child care, and an active stance against any form of sex discrimination or subordination. With respect to geographical distribution, emphasis should be placed on recruitment of students from marginalised areas, offering financial and career incentives to providers serving these areas, and deploying the power of IT to ease professional isolation.
8. Expansion from academic centres to academic systems, extending the traditional discovery-care-education continuum in schools and hospitals into primary care settings and communities, strengthened through external collaboration as part of more responsive and dynamic professional education systems.
9. Linking together through networks, alliances, and consortia between educational institutions worldwide and across to allied actors, such as governments, civil society organisations, business, and media. In view of faculty shortages and other resource constraints, every developing country is unlikely to be able to train on its own the full complement of health professionals that is required. Therefore, regional and global consortia need to be established as a part of institutional design in the 21^st^ century, taking advantage of information and communication technologies. The aim is to overcome the constraints of individual institutions and expand resources in knowledge, information, and solidarity for shared missions. These relations should be based on principles of non-exploitative and non-paternalistic equitable sharing of resources to generate mutual benefit and accountability.
10. Nurturing of a culture of critical inquiry as a central function of universities and other institutions of higher learning, which is crucial to mobilise scientific knowledge, ethical deliberation, and public reasoning and debate to generate enlightened social transformation.
