# Supplementary material for: Developing an evaluation approach for the in-depth review of a new undergraduate medical programme as a complex system
Source: PLoS One. 2024 Dec 31;19(12):e0312730. doi: 10.1371/journal.pone.0312730 (PMC11687765; doi:10.1371/journal.pone.0312730)
Supplement: S3 Text — (DOCX) [file pone.0312730.s003.docx]

| **MBBS Review Steering Committee 2015**  Faculty of Medicine, University of Botswana | 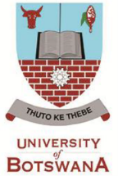 |
| --- | --- |

**10. Review questions and data sources**

NOTE:

- I = interviews; Q = questionnaires; D = documents; O = observations; F = focus group discussion
- Whenever possible questionnaires will be used rather than interviews – data easier to analyse. Where necessary questionnaire data can be clarified/ enriched by short interviews. Interview and focus group data will be written down, not recorded.
- For some questions good data are not easily available. In such cases they will be collected from different sources for triangulation.
- ‘Detailed curriculum documents’ includes block/ rotation guides and prepared student study materials (hard copy or online). These will have to be sampled according to the nature of each question.
- Students will be sampled according to the nature of the question
- For each question the data will be compared to the standards agreed upon, as set out in the documents specifying these.

| **Review questions** | **Data sources** |
| --- | --- |
| - 1. To what degree are the FoM and the MBBS programme autonomous?   2. How well are we partnering with stakeholders in planning and implementation? (MoH, BHPC, community). In the case of the community, which individuals or groups exactly are the real partners? How are their felt and expressed needs determined and taken into account?   3. What is the effect on teaching of the lack of a joint agreement with MoH?   4. Is FoM training doctors for the national health system?   5. How well is FoM and its MBBS programme meeting current and projected future national needs at different levels?   6. How suitable are UB rules and regulations for the MBBS programme? (e.g. SECAT, reporting deadlines, timing of exams); are there discrepancies which negatively affect the MBBS programme? | - 1. Dean, Faculty administrator, FoM academics, DVCFA and DVCAA (I); UB policy documents (D)   2. Dean and senior academics, BHPC and MoH managers, community members identified (I/Q); records of meetings (if any) (D)   3. Dean, clinical HoDs, hospital superintendents, students (I)   4. Dean, senior academics, BHPC and MoH managers (I/Q); detailed curriculum documents, MoH and MoE documents (D)   5. BHPC, MoH, HRDC managers (I)   6. Senior academics, HoDs, Faculty administrator, DVCAA, CAD managers, students (Q/I); policy documents and documents for use (D) |
| - 1. Is the FoM VMV statement adequate – does it give a detailed description of the MBBS end product, mention FoM’s chosen educational strategy, deal with community health needs and health care system needs and social accountability? Does it make reference to research attainment and global health? Did FoM consult with stakeholders in preparing it and do stakeholders have access to it?   2. About the MBBS programme/ curriculum model:      1. Is it defined? Is it outcome-based and competency-based?      2. Does it describe the content and sequencing of courses and other elements to ensure coordination? Is horizontal and vertical integration achieved? (integration between Phase I and Phase II, as well as integration within phases)      3. Are core and elective elements defined and balanced?      4. Are steps taken to prevent content overload? Is the material that is presented relevant to medical practice in Botswana?      5. Are instructional methods defined?      6. Is skills training/ patient contact structured rationally from Year 1 until the end? Is there sufficient patient contact, is there specified time in major clinical disciplines? Is there attention to patient safety?      7. Is the programme truly community-based? Is CBE patchy or is it integrated in the whole programme? What influence does it have? What is its quality?      8. Does FoM practise ‘interdependence in education’ in the programme? are there inter-professional learning events? If so, which other professions are either as teachers or students working with the medical students? What opportunities are there for such education?      9. Does it balance global principles with the local context? Is it locally relevant but also uses international resources?      10. Does FoM practise ‘transformative education’ in the programme? are there opportunities for leadership/ management skills learnt and practised?      11. Does it enshrine equality? Does it promote service in underserved areas? Is it culturally authentic?      12. Does it intend students to be responsible for their own learning and provide explicit opportunities for this to happen?      13. Does it prepare students for future roles and careers, for lifelong learning?   3. Programme outcomes: do these include/ define/ prepare for      1. All knowledge, skills and attitudes needed in: - biomedical sciences (also the latest and anticipated future needs and developments); concepts/ skills on which to base clinical science; there is a minimum package of skills to be learnt - behavioural and social sciences, medical ethics, medical jurisprudence (also up-to-date and forward-looking) - clinical practice after graduation (also up-to-date and forward-looking); management of priority causes of morbidity and mortality - practical health promotion and disease prevention; communication/ advocacy   - 1. Exposure to Emergency Medicine and Family Medicine (working with undifferentiated patients in clinics); if it is insufficient are other clinical disciplines willing to give up some of their time for this purpose?     2. Interface with complementary (and traditional) medicine     3. Global/ international health     4. The scientific method: analytical skills and critical thinking, information literacy and retrieval, applying EBM and EB policies, research (methods and practice)     5. Community health needs, health care system needs, social accountability     6. Professionalism and ethics     7. Being leaders and managers of their own practices, resources and health services   1. Are the outcomes/ objectives at different levels of the programme written correctly? Do they indicate the depth to which students are expected to engage with them?   2. Is the possibility of an intercalated degree between Phase I and Phase II feasible?   3. Does the design of the whole curriculum (in the broadest sense) support the development of a graduate who can perform the roles set out in the SoM, BHPC and AfriMEDS documents? | - 1. Latest version of FoM VMV document (D) original academic staff, academic staff in general, students, stakeholders who should have been consulted (I)      1. Original MBBS programme; detailed curriculum documents (D)   2.2.2 Detailed curriculum documents (D); senior academics, block and rotation coordinators (I)   - - 1. Detailed curriculum documents (D)     2. Detailed curriculum documents; morbidity/ mortality data for Botswana (D)     3. Detailed curriculum documents (D)     4. Detailed curriculum documents (D); current skills training programme in Phase 1 (D); clinical HoDs, senior students (Q/I)     5. Detailed curriculum documents (D); senior staff in Phase 1, Public Health and Family Medicine; students (Q/I); site visits to placement venues (O,I)     6. Detailed curriculum documents (D); block and rotation coordinators (Q/I); programme coordinators in FoHS (I)     7. Detailed curriculum documents (D); block and rotation coordinators (Q/I)     8. Detailed curriculum documents (D); block and rotation coordinators, students (Q/I)     9. Detailed curriculum documents, admissions policy (D); block and rotation coordinators, students (Q/I)     10. Detailed curriculum documents (D); block and rotation coordinators, students (Q/I)     11. Detailed curriculum documents (D); block and rotation coordinators, students (Q/I)     12. Detailed curriculum documents; latest MoH health statistics (D); HoDs, block and rotation coordinators, students (Q/I)     13. Overall Phase I and II plans (D); clinical skills programme for Phase I (D); clinical HoDs (I); students (Q)     14. Block and rotation coordinators, students (Q/I); detailed curriculum documents (D)     15. Block and rotation coordinators, students (Q/I); detailed curriculum documents (D)     16. Detailed curriculum documents, research outputs with student participation (D); block and rotation coordinators, FoM librarian, clinical tutors, students (Q/I); bedside teaching and PBL (O)     17. Detailed curriculum documents (D); block and rotation coordinators, students (Q/I)     18. Detailed curriculum documents, external examiner reports (D); block and rotation coordinators, clinical tutors, students (Q/I); bedside teaching (O)     19. Detailed curriculum documents (D); block and rotation coordinators, students (Q/I)   1. Detailed curriculum documents (D); block and rotation coordinators (Q/I)   2. Dean Faculty of Science; senior academics (I/F)   3. Detailed curriculum documents (D); senior academics, block and rotation coordinators (I/F); interns and interns supervisors (Q) |
| In considering the resources the underlying question is: are they conducive to/ supportive of learning?   - 1. Does FoM have and use a staff recruitment and selection policy which describes types of staff and the balance between them, their responsibilities, evaluation criteria, and relates to the VMV statement and economic issues?   2. Are teachers transformed, positive examples as agents-of-change? Are they good models of professional behaviour and ‘soft skills’? How adequate is their cultural understanding and language ability? Do they promote a culture of critical enquiry and public reasoning/ debate?   3. Does FoM have a use a staff ‘activity and development’ policy which balances teaching/research/service, sets staff:student ratios, recognises merit and sets standards for promotion, promotes use of clinical work for research/ teaching, ensures staff is up-to-date, includes training (e.g. in education and research) and support?   4. Are physical facilities adequate for curriculum delivery (including space for self-study, transport for Phase 2 students to hospitals etc.)? Are there plans to rectify shortcomings?   5. Where are students accommodated? Are the accommodation facilities adequate for studying?   6. Is the training platform a teaching health system including primary care and community settings? Are students being trained in environments where they will be most needed? Are admission rates and student:patient ratios adequate?   7. Does the learning environment (in its broadest sense) support students, staff, patients and the curriculum? Does it embody equity and non-discrimination? How do students experience the environment?   8. How is the study of individual students funded? Is such funding adequate?   9. Are clinical training resources sufficient in terms of patient numbers, facilities and equipment, supervision? Are these being adapted to best serve the community? (note considerable overlap with 3.4; the two are really one item)   10. Does FoM have a policy regarding use and evaluation of ICT in its programmes? Do teachers and students use ICT for independent learning, accessing information and patient management? How adequate are ICT services for students and staff? (including WiFi).   11. Does FoM have and use a policy to promote the link between research and education? Is the curriculum based on scholarship and research? Are research priorities made clear? Does research influence current teaching?   12. Does FoM have a policy on the use of educational expertise for curriculation, teaching and assessment, and does it have access to such expertise and does it use it? Does it develop staff expertise in education and promote educational research?   13. Does FoM have and use a policy regarding national and international collaboration with other educational institutions, and transfer or educational credits? What has the contribution of such partners to the MBBS programme been to date? Does it facilitate rational and ethical staff and student exchanges, and form and use links in the form of networks, alliances and consortia between educational institutions?   14. Has FoM defined its governance structures and functions? Does this define its relationship with UB, committee structure, student and stakeholder representation? Are its operations transparent?   15. Has FoM described the role/ responsibilities of its academic leadership in managing the MBBS programme? Does it evaluate how effective this is from time to time?   16. Does FoM have a process for and authority to resource the MBSB programme – a dedicated educational budget? Does it allocate the resources appropriately and does it have the authority to do so? Are resources allocated to support scientific development and health needs? What are the sources of the MBBS budget?   17. Is the FoM’s administrative structure and staff adequate to support the educational programme and ensure good general and resource management (e.g. help for admissions, BHPC registration)? Does it operate a system of regular internal quality assurance? Is it responsive to staff and students?   18. How adequate is the system of storage of programme information and documentation? Is it dependable and sustainable? Is there a system for following up the career path of MBBS and MMed graduates?   19. Does FoM have regular and constructive interaction with MoH and other health-related stakeholders? Is such interaction formalised in agreements? | - 1. Dean, HR manager, HoDs (I); documents relating to staff recruitment (D)   2. Dean, senior and junior academics, HoDs, students (Q,F); bedside teaching (O)   3. Dean, HR manager (I); academic and administrative staff (Q); documents relating to staff administration and deployment (FoM and UB e.g. PMS) (D)   4. Block and rotation coordinators, HoDs, students; Clinical Skills Coordinator (Q); facilities in Gaborone and elsewhere (O)   5. Students (Q); student accommodation (O)   6. Senior academics, rotation coordinators (Q/I); data on admissions and occupancy rates (D)   7. Academic and administrative staff, students (DREEM tool) (Q)   8. Dean, Dr J Masunge, DVCFA, FoM administrator (I), students (Q)   9. Clinical HoDs, Phase II students,   10. Policy document (D); academic and administrative staff, students (Q); CAD/EDUTECH and UB Library staff, FoM IT manager (I)   11. Dean (I); FRPC members (F); documents related to research (D); block and rotation coordinators (Q); academic staff (Q)   12. Dean and senior academics (Q/I); DME staff and associates (F); academics (Q)   13. Dean, senior academics, HoDs, managers/ directors of partner groups: BoMEPI, UPenn, Harvard groups, etc. (Q/I); UB policy on international relations; MoA documents with MEPI, UPenn, Harvard, MoH, BHPC, CONSAMS (D)   14. Dean, senior academics; FoM HR manager and Administrator (Q/I); FoM and UB documents relating to governance (D)   15. Dean, senior academics (Q/I); relevant documents (D)   16. Dean, FoM administrator, FoM purchase/ procurement officer, DVCFA, HoDs (Q/I); relevant documents (FoM budgets, UB financial policies) (D)   17. Dean, FoM administrator, DME members, HoDs, academic and administrative staff, students (Q/I/F)   18. Dean, senior academics, HR manager, FoM IT manager, block and rotation coordinators (F/Q/I)   19. Dean, BPHC managers, MoH managers, MIT group managers (I); senior academics (F); minutes of meetings, original MBBS submission document, MoA documents (D) |

| - 1. Does FoM have an MBBS curriculum committee which effectively manages the MBBS programme, innovates when needed, and has a membership representative of staff, students and other stakeholders? Does it seek inputs from the environment where graduates work (including communities) and make appropriate changes to the programme?   2. How similar are the written/official, taught and assessed curricula?   3. Does the MBBS programme have clear links to practice and training after graduation?   4. About the teaching methods used in the programme:      1. Are the teaching methods that are used appropriate to the domain and level of the learning objectives that they are implementing?      2. Is the quality of teaching in the different domains in different blocks and rotations of a high standard? (including PBL, plenaries, skills training, teaching professional attitudes)      3. Are clinical decision making, professional development and ethics taught and learnt in appropriate ways?      4. Is the content of PBL cases sufficiently ‘local’?      5. Are Anatomy teaching strategies in Phases 1 and 2 effective?      6. Does teaching of history taking and the consultation give students sufficient cultural sensitivity?   5. About the materials used to support teaching/ learning in the programme:      1. Is the quality of teaching/ learning aids and materials of a high standard? (PowerPoints, hand-outs, references, checklists etc.)      2. To what extent are electronic teaching/ learning resources being used?   6. About assessment in the programme:      1. Does FoM state the principles, methods and practices used for assessment, including pass/fail criteria, rules for re-examination etc.?      2. Why does the current assessment system seem unable to distinguish between high and low achievers, to identify students who should fail? What is the range of marks in written and OSCE exams?      3. Does assessment cover all domains of learning using a range of appropriate methods? (constructive alignment). Is professionalism adequately assessed?      4. What is the quality of current assessment methods (MCQs, SAQs, OSCEs, miniCEXs etc.)?      5. What is really being assessed? Does assessment promote deep learning and integration of learning?      6. Is there a system to evaluate the validity and reliability of assessment? Is the current way of allocating marks for MBBS V OSCEs valid?      7. Are blueprinting and standard setting done expertly and consistently?      8. Is assessment checked by external examiners?      9. How are pass/ fail decisions made? Is there a system for students to appeal assessment results?      10. Is there an appropriate balance between formative and summative assessment?      11. Is assessment actively used to guide evaluation of and decisions about students’ academic progress?      12. Is the number and type of assessments planned to maximise the type of learning required?      13. Is regular and fair feedback given after assessments?   7. About student admission to the programme:      1. Does FoM have and use a policy on admission, operating fairly and spelling out the process of admission? Is admission of disabled students spelt out? Is there a policy which allows transfer of students from other programmes and graduate student admissions?      2. Does the admission policy promote the presence of doctors in underserved areas? Are students from marginalised areas and populations selected? Is there a geographical component to selection?      3. Is the size of the student intake specified and related to educational resources? What is the evidence supporting the current intake number? Is the size of intake reviewed from time to time?   8. Does FoM have a system or unit which offers comprehensive support to students, dealing with a range of problems and needs? Is this resource adequately funded and does it operate with full confidentiality? Does it monitor student progress?   9. Does students’ English language ability affect their studies negatively?   10. Does FoM have and implement a policy on student representation in all relevant bodies concerned with the MBBS programme? Does it encourage and facilitate participation in student activities and organisations?   11. Does FoM have a quality assurance system routinely monitoring all aspects of the MBBS programme, including student progress? Is staff and student feedback on relevant aspects regularly sought? Is the feedback of other stakeholders regularly sought? Are the findings of quality monitoring reported to all relevant stakeholders? Do findings lead to relevant changes and developments in the programme?   12. Does FoM conduct periodic comprehensive evaluations/ reviews of the MBBS programme? Are resources available for such reviews? Do the findings lead to concerted action for improvement, in terms of policies and practices? Does the programme readily adapt to the evolving roles of doctors?   13. Does FoM have a clearly articulated policy on educational research, and is this leading to data which is fed back into the programme? Is enough educational research being done on Phase 2 of the programme? | - 1. Senior academics, Phase 1 and 2 coordinators with block and rotation coordinators (i.e. the Phase 1 and 2 committees) (I)   2. Detailed curriculum documents, PBL case documents, notes/ PPs used by teachers, handouts and references for students (hard or soft copy), exam papers for Phase 1 and 2 (D)   3. Senior academics, clinical HoDs, Phase 1 HoDs and subject heads, MITP staff, BHPC and MoH managers (Q/I), MIT programme plan (D), intern preparedness research report (D)      1. Detailed curriculum documents, analysis of block/ rotation study guides, session plans/ notes used by teachers (D)      2. Session plans/ notes used by teachers (D), teaching sessions (O)      3. Clinical and basic science teachers (Q), teaching sessions (O)      4. PBL case documentation (D)      5. Phase 1 Anatomy teachers (I), FoM clinical teachers (junior and senior), students Phase 1 and 2 (Q)      6. Skills teachers in Phase 1, clinical teachers, students (Q/I); bedside teaching (O)      7. Lesson plans/ notes, PowerPoints, hand-outs, references, checklists from all the blocks and rotations (D); Block and rotation coordinators (Q)      8. Phase 1 HoDs/ discipline heads, Phase 2 HoDs/ sub-discipline heads (Q)      9. FoM policy documents (e.g. academic calendar) (D)      10. Block and rotation coordinators (Q/I); mark sheets for the past 5 years (D)      11. Block and rotation coordinators (Q/I); Written exam/ test papers, marking sheets for practical tests/ exams, other CA documents (D)      12. Written exam/ test papers, marking sheets for practical tests/ exams, other CA documents (D); observing practical exams (O)      13. Block and rotation coordinators, students (Q/I); Written exam/ test papers, marking sheets for practical tests/ exams, other CA documents (D)      14. Block and rotation coordinators, Phase 2 coordinator, students (Q/I)      15. Block and rotation coordinators (Q/I); exam/ test/ OSCE blueprints, standard setting documentation (D)      16. Block and rotation coordinators (Q/I); external examiner reports (D)      17. ~~Dean~~, senior academics, students (Q/I), FoM/ UB regulations (e.g. academic calendar, UB policy on assessment) (D)      18. Block and rotation coordinators (Q/I); Written exam/ test papers, marking sheets for practical tests/ exams, other CA documents (D)      19. Block and rotation coordinators, students (Q/I)      20. Block and rotation coordinators (Q/I)      21. Block and rotation coordinators, students (Q/I)      22. Admissions Committee (F); admissions policy (D)      23. Admissions Committee (F); admissions policy, student biographical data (D)      24. Dean, senior academics (Q/I); Admissions Committee (F); admissions policy (D)   4. Dean, senior academics, junior academics, Phase 1 and 2 HoDs and subject heads (Q/I)   5. Junior academics, Phase 1 and 2 HoDs and subject heads, students (Q)   6. Dean, senior academics, student leaders (Q/I)   7. Senior academics, block and rotation coordinators, students, BHPC, MoH, HRDC, CAD, ADO managers (Q/I), relevant documents/ reports (D) NOTE: these documents to be discussed with the persons above   8. Dean, senior academics, MoH, BHPC, ADO and CAD directors (Q/I), relevant documents/ reports (D) (NOTE: the Leinster report exists for analysis and respondents will be asked for others, to discuss with them)   9. Dean, senior academics, Phase 1 and 2 HoDs and subject heads (Q/I); FRPC members (F) educational research policy (D) (NOTE: will be obtained from those above if it exists and discussed with them) |
| --- | --- |

| - 1. Does FoM analyse the performance of cohorts of students and graduates in terms of its mission, intended outcomes/ curriculum, and student admission biographies?   2. Does FoM make the outcomes of such analysis available to relevant committees or units in the FoM (e.g. admissions, curriculum committee, student support services) and does this information result in meaningful changes when necessary?   3. What is the level of competence of graduates as interns in important areas such as: - Quality clinical care of patients: skilled, motivated, patient-centred, productive, ethical, scientific - Lifelong/ ongoing learning and critical thinking - Ability to work effectively at all levels of the health service, as members of the health care team - Innovation, social responsibility, independent action, confidence - Safe practice   1. To what extent are graduates in intern positions carrying out the seven roles of the physician effectively?   2. To what extent do the graduates reflect the vision that the SoM had when the programme started?   3. Have any graduates been involved in doing research? If so in what kinds of research? | - 1. Dean, senior academics, HoDs, block and rotation coordinators (Q/I), relevant documents/ reports (D)   2. Admissions Committee, Phase I and II coordinators, Dean, senior academics (Q/I)   3. UB graduate interns, their supervisors (Q/I/F) (NOTE: the ‘intern preparedness’ research provides the necessary information)   4. UB graduate interns, their supervisors (Q/I/F) (NOTE: the ‘intern preparedness’ research provides the necessary information)   5. Clinical HoDs (I/F); FoM VMV document (D)   6. Clinical HoDs, Phase 1 HoDs, UB graduate interns, intern supervisors (Q/I) |
| --- | --- |
| - 1. What effect does the presence of students in health facilities have on the level of care? (the effect on doctors and other health workers practising in the facilities; the effect on patient care and wellbeing)   2. How effective is the intern system within which the graduates are operating?   3. How effectively are interns managing (in terms of prevention and treatment) the major diseases causing morbidity and mortality in Botswana: HIV infection, TB, cardiovascular disease, cancer, trauma, diarrhoeal disease, ARIs, perinatal and maternal mortality, child malnutrition, anaemia, diabetes?   4. What is the opinion of patients, their families and their communities about the quality of care they receive from UB’s medical graduates?   5. To what extent are UB’s medical graduates serving/ prepared to serve in underserved areas and communities? | - 1. Clinical HoDs, hospital superintendents (Phase 2) managers in PHC clinics (Phase 1) (Q/I) (NOTE: these data are available in research done at Maun and Mahalapye – not at PMH)   2. UB graduate interns, intern supervisors, clinical HoDs in PMH, MITP managers (Q/I)   3. UB graduate interns, their supervisors, clinical HoDs in PMH (Q/I)   4. Patients, their families and placement supervisors from the community in clinical settings used by students (I/F)   5. UB graduate interns (Q); research report M Kebaetse et al (D) (NOTE: these data are available) |
