# Supplementary material for: Developing an evaluation approach for the in-depth review of a new undergraduate medical programme as a complex system
Source: PLoS One. 2024 Dec 31;19(12):e0312730. doi: 10.1371/journal.pone.0312730 (PMC11687765; doi:10.1371/journal.pone.0312730)
Supplement: Attachment — Submitted filename: Response to Reviewers (1).docx [file pone.0312730.s004.docx]

Developing an evaluation approach for the in-depth review of an undergraduate medical programme as a complex system

| **No.** | **Reviewers’ Comments** | **Authors’ feedback** |
| --- | --- | --- |
|  | **General Comments** | |
| 1. | Is the manuscript technically sound, and do the data support the conclusions?  The manuscript must describe a technically sound piece of scientific research with data that supports the conclusions. Experiments must have been conducted rigorously, with appropriate controls, replication, and sample sizes. The conclusions must be drawn appropriately based on the data presented.  **Feedback: Partly** | We thank the reviewer for this comment. Our manuscript is reporting an example of evaluation research: the evaluation of an entire five-year educational programme. From the results of our study, we propose a framework to guide the evaluation of educational programmes as complex systems. This manuscript was therefore submitted under the PLOS ONE category of ‘Developing new methods/ tools’. |
| 2. | Has the statistical analysis been performed appropriately and rigorously?  **Feedback: Yes** | We thank the reviewer for this encouraging comment. |
| 3. | Have the authors made all data underlying the findings in their manuscript fully available?  **Feedback: No** | We thank the reviewer for this comment. The reasons for not implementing this requirement are given in line 17 below. |
| 4. | Is the manuscript presented in an intelligible fashion and written in standard English?  PLOS ONE does not copyedit accepted manuscripts, so the language in submitted articles must be clear, correct, and unambiguous. Any typographical or grammatical errors should be corrected at revision, so please note any specific errors here.  **Feedback: Yes.** | We thank the reviewer for this encouraging comment. |
| 5. | The University of Botswana medical school was established in 2009 and the program evaluation was performed in 2016. Please consider deleting "new" from medical program in the title. | We thank the reviewer for this comment. We have removed ‘new’ from the title. |
| 6. | Have you performed a more recent program evaluation? If yes, did you use the same theoretical framework and evaluation model? What was the outcome of the recent evaluation compared to 2016? | The second review has not been carried out; however the approach to programme evaluation described in the manuscript will inform the next review. |
|  | **Background** | |
| 7. | The literature review provides an extensive and robust discussion of multiple theories and paradigms in program evaluation. However, this discussion is better suited as a scoping review on this topic. Please consider a succinct description of the general and contextual reasons for a program evaluation, the most frequently applied theoretical framework in program evaluation - including examples of institutions that have applied this framework successfully and why it was chosen. | - We thank the reviewer for these comments. - We have revised this section to make it more succinct and focused, also structuring it more clearly around the following: reasons for program evaluation (67-71), and frequently applied theoretical frameworks and models applied in program evaluation (89-121). In lines 127-131 reference is made to a scoping review of 44 studies reporting evaluations based on complexity theory, so this is not a new phenomenon; however none of those we located were evaluations of whole programmes. - The rationale for our using this model is given in lines 123-127. |
| 8. | You also provided a broad description of evaluation models. Please consider narrowing your discussion by summarizing these models and providing a rationale for the model you used for your program evaluation. |  |
| 9. | What are your SMART objectives for this evaluation process? | Thank you for the question. We had one broad objective for the study. To address the broad objective we then developed 90 specific objectives (phrased as questions), each of which we believe is SMART.   - *Broad objective:* To review the quality of the MBBS programme at the University of Botswana as it is currently implemented (plan, process and product) in relation to its initial aims and national and international standards. - *Specific objectives for this proposal*   For convenience the 90 objectives/ questions are appended in a Supporting Information document (S3 Text.Questions, data sources, instruments) |
|  | **The Approach** | |
| 10. | Lines 183 - 185 don't add any relevant information to this manuscript. | We thank the reviewer for this comment. We revised the statement to focus on the context of the programme, as follows – The University of Botswana Faculty of Medicine (UB FoM) offers a five-year, hybrid PBL undergraduate Bachelor of Medicine, Bachelor of Surgery (MBBS) curriculum (148-149) |
| 11. | Please provide the 90 evaluation questions that were derived from the evaluation standards as an appendix. | We thank the reviewer for this comment. They are provided in a Supporting Information document (S3 Text.Questions, data sources, instruments) |
| 12. | What criteria did you use to determine the appropriate data collection instrument for stakeholders? | We thank the reviewer for this comment. The nature of each question directed us to the data and then the data sources that we needed to achieve the answers to the question. The instruments used were selected according to (1) the data needed and the (2) sources from which the data could be obtained, so these two were our ‘criteria’ for selecting instruments (see S3 Text.Questions, data sources, instruments). In summary:   - Interview guides: for obtaining qualitative data from individuals - Focus group discussion guides: for obtaining qualitative data from groups of individuals whose interaction would be likely to enrich the data - Questionnaires: for obtaining quantitative as well as simple qualitative data from numbers of individuals, so that they could be summarised. - Observation guides: to guide the observation of activities in a structured way to provide information needed for particular study questions - Document study guide: see response below.   We followed the principle of triangulation throughout. An example is given in Table 4 – for that question seven data sources were used, each with appropriate instruments. |
| 13. | What is the purpose and content of the document study guide? | We thank the reviewer for this comment. By ‘document study guide’ we meant instruments which guided our reading of each document that had been identified as a data source for various research questions (e.g. when reading the MBBS Admissions Policy we had to look for information needed to answer 4 of the study’s research questions and the instrument guided us to do that). In the literature this is also called a ‘document analysis guide’. |
|  | **Validation** | |
| 14. | The introduction of Cilliers's ten characteristics of a complex system later in your manuscript poses another challenge in understanding the purpose of your research. Was your goal to validate the complexity theory in your institution's program evaluation? | - We thank the reviewer for this comment. We have now introduced our intention to use Cilliers in the ‘Background’ section (138-140). We also moved the analysis using the ten features of Cilliers from the end of the manuscript, to follow on the ‘six steps’. Our use of Cilliers is also mentioned in the Abstract. - Our goal was to use the features of a complex system (as set out by Cilliers), to validate our six-step approach: did our approach illuminate each of the ten features of this particular complex system? |
|  | **Financial disclosure** | |
| 15. | Please be informed that funding information should not appear in the Acknowledgments section or other areas of your manuscript. We will only publish funding information present in the Funding Statement section of the online submission form. Please remove any funding-related text from the manuscript.  Thank you for stating the following financial disclosure:  "No external funding was received to support this research. Planning workshops, data collection and data analysis expenses were fully funded by the Faculty of Medicine, University of Botswana."  At this time, please address the following queries:  a) Please clarify the sources of funding (financial or material support) for your study. List the grants or organizations that supported your study, including funding received from your institution.  b) State what role the funders took in the study. If the funders had no role in your study, please state:  c) If any authors received a salary from any of your funders, please state which authors and which funders.  d) If you did not receive any funding for this study, please state: “The authors received no specific funding for this work.”  Please include your amended statements within your cover letter; we will change the online submission form on your behalf. | Thank you for this comment. We have removed this information from the manuscript and will present it in the cover letter, as follows:  No external funding was received to support this research. Planning workshops, data collection and data analysis expenses were fully funded by the Faculty of Medicine, University of Botswana from its routine yearly budget. The funding authority in the Faculty of Medicine had no role in study design, data collection and analysis, decision to publish, or preparation of the manuscript. The researchers undertook the work as part of their routine academic duties and did not receive any additional remuneration. The authors (all of whom were researchers) also received no specific funding for this work. |
|  | **Data Related Comments** | |
| 16. | If applicable, we recommend that you deposit your laboratory protocols in protocols.io to enhance the reproducibility of your results. Protocols.io assigns your protocol its own identifier (DOI) so that it can be cited independently in the future. For instructions see: https://journals.plos.org/plosone/s/submission-guidelines#loc-laboratory-protocols. Additionally, PLOS ONE offers an option for publishing peer-reviewed Lab Protocol articles, which describe protocols hosted on protocols.io. Read more information on sharing protocols at <https://plos.org/protocols?utm_medium=editorial-email&utm_source=authorletters&utm_campaign=protocols> | Thank you for this comment. This is not relevant to our study which was not laboratory based. |
| 17. | Did you know that depositing data in a repository is associated with up to a 25% citation advantage (https://doi.org/10.1371/journal.pone.0230416)? If you’ve not already done so, consider depositing your raw data in a repository to ensure your work is read, appreciated and cited by the largest possible audience. You’ll also earn an Accessible Data icon on your published paper if you deposit your data in any participating repository (<https://plos.org/open-science/open-data/#accessible-data>).  In this instance, it seems there may be acceptable restrictions in place that prevent the public sharing of your minimal data. However, in line with our goal of ensuring long-term data availability to all interested researchers, PLOS’s Data Policy states that authors cannot be the sole named individuals responsible for ensuring data access.  Before we proceed with your manuscript, please also provide non-author contact information (phone/email/hyperlink) for a data access committee, ethics committee, or other institutional body to which data requests may be sent. | Thank you for this suggestion. In this case there are restrictions in place that prevent the public sharing of our minimal data. The consent forms we used asked study participants to agree to ‘provide the information requested, which will be incorporated (anonymously) *in the Review Report only’*. We are therefore not able to make these data available without restrictions.  We are following the alternative route you recommend, as follows:  Readers which wish to have access to the data supporting this research must please apply to: The Office for Research and Development, University of Botswana ([research.ethics@ub.ac.bw](mailto:research.ethics@ub.ac.bw)) |
|  | **Other Comments** |  |
| 18. | Your ethics statement should only appear in the Methods section of your manuscript. If your ethics statement is written in any section besides the Methods, please move it to the Methods section and delete it from any other section. | Thank you for this comment. We have moved the Ethics statement to the end of the Methods section (305-317) |
| 19. | Please include captions for your Supporting Information files at the end of your manuscript, and update any in-text citations to match accordingly. Please see our Supporting Information guidelines for more information: http://journals.plos.org/plosone/s/supporting-information | Thank you for this comment. We have done as you suggested for three files and have implemented your ‘Supporting Information’ guidelines |
|  | Please ensure that your manuscript meets PLOS ONE's style requirements, including those for file naming | Thank you for this comment. We have consulted the PLOS ONE ‘Style and Format’ guidelines and have adhered to them. |
| 20. | Reviewer #1: Please review the manuscript for typographical errors. | Thank you for this comment. We have checked the manuscript for typographical errors on several occasions and have addressed them. |
