# Supplementary material for: Developing an evaluation approach for the in-depth review of a new undergraduate medical programme as a complex system
Source: PLoS One. 2024 Dec 31;19(12):e0312730. doi: 10.1371/journal.pone.0312730 (PMC11687765; doi:10.1371/journal.pone.0312730)
Supplement: Attachment — Submitted filename: Responses to reviewers.16 August 2024.docx [file pone.0312730.s005.docx]

**Developing an evaluation approach for the in-depth review of an undergraduate medical programme as a complex system**

Please note that the line numbers below refer to the clean copy of the manuscript. We have also highlighted the required changes in the clean copy.

| **No.** | **Reviewers’ Comments** | **Authors’ feedback** |
| --- | --- | --- |
|  | **General Comments** | |
| 1. | Is the manuscript technically sound, and do the data support the conclusions?  The manuscript must describe a technically sound piece of scientific research with data that supports the conclusions. Experiments must have been conducted rigorously, with appropriate controls, replication, and sample sizes. The conclusions must be drawn appropriately based on the data presented.  Reviewer #1: Yes  Reviewer #2: Yes | We thank the reviewer for this encouraging comment. |
| 2. | Has the statistical analysis been performed appropriately and rigorously?  Reviewer #1: N/A  Reviewer #2: N/A | We thank the reviewer for this encouraging comment. |
| 3. | Have the authors made all data underlying the findings in their manuscript fully available?  The [PLOS Data policy](http://www.plosone.org/static/policies.action#sharing) requires authors to make all data underlying the findings described in their manuscript fully available without restriction, with rare exception (please refer to the Data Availability Statement in the manuscript PDF file). The data should be provided as part of the manuscript or its supporting information, or deposited to a public repository. For example, in addition to summary statistics, the data points behind means, medians and variance measures should be available. If there are restrictions on publicly sharing data—e.g. participant privacy or use of data from a third party—those must be specified.  Reviewer #1: No  Reviewer #2: No | Thank you for this suggestion. In this case there are restrictions in place that prevent the public sharing of our minimal data. The consent forms we used asked study participants to agree to ‘provide the information requested, which will be incorporated (anonymously) *in the Review Report only’*. We are therefore not able to make these data available without restrictions.  We are following the alternative route you recommend, as follows:  Readers which wish to have access to the data supporting this research must please apply to: The Office for Research and Development, University of Botswana ([research.ethics@ub.ac.bw](mailto:research.ethics@ub.ac.bw)) |
| 4. | Is the manuscript presented in an intelligible fashion and written in standard English?  PLOS ONE does not copyedit accepted manuscripts, so the language in submitted articles must be clear, correct, and unambiguous. Any typographical or grammatical errors should be corrected at revision, so please note any specific errors here.  Reviewer #1: Yes  Reviewer #2: Yes | We thank the reviewer for this encouraging comment. |
| 5. | Reviewer #1: Background:  • The authors have provided a detailed overview of program evaluation and systems theory. The information provided is beneficial; however, conciseness is needed.  • I would suggest trimming the background to provide readers with concise information supported by recent theories and findings.  • One suggestion to make the background concise is to provide supporting visual representation instead of text.  • For systems theory, will it be possible to provide some visuals of complex systems? Showing interaction between systems components, interaction with the surroundings etc. will be helpful for readers to grasp the complex idea in a concise manner. | We thank the reviewer for these comments.  In response to the previous round of feedback we revised the background to make it more succinct and focused – we in fact trimmed it from 5½ pages (1413 words) 3½ pages (894 words) – this was in spite of also having moved discussion of Cilliers’s model from a later section of the document to the Background. We also structured the Background more clearly around the following: reasons for program evaluation (66-71), scope of evaluation (71-75), data collection methods used (75-80), standards of evaluation used (82-87), and frequently applied theoretical frameworks and models applied in programme evaluation (89-121). This last section is more detailed because the issue of theory is at the heart of this work and needed to be explored thoroughly.  We are concerned that further trimming of the introduction may compromise our overall argument. In the literature review we sought to provide evidence that our evaluation is unique in terms of its combination of scope, methods and theoretical grounding. |
| 6. | • Several citations are old. For example, starting in line 100, the authors have a whole paragraph discussing the relationships between the elements and their contributions to the system. The key citation in this paragraph (Von Bertalanffy, 1968) is from 1968!  • Several citations are outdated. For example, Prigogine, 1997 and Wakeford, 1980. Please provide updated references if possible.  Reference # 3, 25 and 32 are too old. Please replace with updated literature. | We thank the reviewer for this comment.   - References 3 has been changed to a similar more recent one. - References 25, 29, 30, 32 and 45 are from seminal original literature referring to models, theories and frameworks that form an important part of the discussion – Prigogine, Cilliers, Von Bertlanffy, Stufflebeam, Mezirow are all internationally respected as initiators of valuable concepts and methods |
| 7. | • In the background, provide information on the context of your program development. Why was developing/introducing a new program necessary? | We thank the reviewer for this comment.  We have inserted a statement of the rationale for this programme under ‘Context’ – line 149-151 |
| 8. | • Line 177: The authors wrote: “The team planning the review unanimously decided that we needed to understand the MBBS programme in its ‘wholeness’ [25]. We realised that..” It is unclear who the planning team is and who the ‘we’ are. Clarify how many authors were part of the planning team and how many of the authors were on the steering committee. • Who was invited to the workshop? Faculty only? Staff? Please mention them in the text. | We thank the reviewer for this comment.  The position of the authors at the different stages of the evaluation has been clarified in lines 165-166.  Attendance at the workshop is set out in lines 161-162 and 163-164. |
| 9. | • In Table-1, the column ‘Originator’ is difficult to comprehend. Consider using ‘Guiding Evaluation Models’ or something similar. | We thank the reviewer for this comment. The correction from ‘Originator’ to ‘Guiding Evaluation Models’ in Table 1 has been made (line 200). |
| 10. | • In Table-2, add a column showing which instrument was used to answer these questions. For example, focus groups, surveys, interviews, attendance, student performance etc. • In the appropriate location in the main text, also mention why one instrument was chosen over another for different questions. | We thank the reviewer for this comment. The column ‘Instruments used to collect data’ has been added to Table 2 (line 234). |
| 11. | • Line 265: Authors mentioned: “We familiarised ourselves with the data, identified emerging themes, and coded them under two headings: ‘achievements’ and ‘issues to consider/areas for improvement/challenges’.” Where are these results? The way it is presented in table 4 is not sufficient. Please provide the subthemes and representative quotes in a separate table. | We thank the reviewer for this comment. In this research we did not intend to conduct inductive qualitative analysis. The pragmatic process we used is the following:   - We noted the question and its number. - Using a prepared guide which listed the instruments providing data for each question, we located all the data provided for that question - We considered all of these data together and looked for recurring patterns, enabling us to list ‘achievements’ and ‘issues to consider/ areas for improvement/ challenges’ related to that question.   The process we followed therefore did not employ ‘subthemes’ and ‘representative quotes’. |
| 12. | • The authors mentioned: “We considered each question as a free-standing unit and brought all the data (qualitative and quantitative) about it together.” Please show these results in graphs. Put the survey questionnaire in the supporting materials. • Provide a flowchart or diagram outlining the timeline and the major steps of this program evaluation. | We thank the reviewer for this comment. However the focus of the paper was not to demonstrate the rigour of the data analysis (which we believe was rigorous, as explained above) but rather to demonstrate the approach followed – triangulating multiple sources of data as a way of viewing our particular complex system appropriately.  As explained above the results were recorded narratively and not graphically.  The research used 96 instruments most of which provided data for several questions – only 10 of these were questionnaires. Most instruments were used several times and between them provided 320 separate pieces of data. For pragmatic reasons it is not feasible to append all of these as supporting materials.  A diagram has been provided at line 179. |
| 13. | Line 22: As you have removed the term ‘new’ from the title, please remove ‘new’ from the abstract as well. | We thank the reviewer for this comment. The change has been made. |
| 14. | Punctuation (.) is missing in line 77. | We thank the reviewer for this comment. This has been corrected. |
| 15. | Line 111: processes can written as ‘processes’ to match with ‘things.’ | We thank the reviewer for this comment. Complexity theory makes a particular distinction between ‘processes’ and ‘things’. To make this change would modify the sense of complexity theory. |
| 16. | Line 130: rewrite the sentence for clarity. A suggestion: The reported applications of the theory included making conceptual frameworks and planning data analysis and interpretation. | We thank the reviewer for this comment. This change has been made. |
| 17. | In paragraph starting at line 156: Use passive voice. Suggestion: ‘They elected a Steering Committee’ can be A steering committee was elected. | We thank the reviewer for this comment. This change has been made. |
| 18. | Line 420: should be ‘spelled’ | We thank the reviewer for this comment. We have made the change. |
| 19. | Update supporting information section with the list of all documents: Information about the ethics approval letters are missing in this section. | We thank the reviewer for this comment.  Under point 12 above we mentioned that there are 320 documents containing data. We respectfully suggest that the information needed to grasp the scope of the review is given in Table 3, so a list of all the documents would be redundant.  Information about the ethics approval letters is given in lines 298-302. |

**The reviewers’ comments and our authors’ feedback after the initial round of review are given below, for reference**

| 1. | Is the manuscript technically sound, and do the data support the conclusions?  The manuscript must describe a technically sound piece of scientific research with data that supports the conclusions. Experiments must have been conducted rigorously, with appropriate controls, replication, and sample sizes. The conclusions must be drawn appropriately based on the data presented.  **Feedback: Partly** | We thank the reviewer for this comment. Our manuscript is reporting an example of evaluation research: the evaluation of an entire five-year educational programme. From the results of our study, we propose a framework to guide the evaluation of educational programmes as complex systems. This manuscript was therefore submitted under the PLOS ONE category of ‘Developing new methods/ tools’. |
| --- | --- | --- |
| 2. | Has the statistical analysis been performed appropriately and rigorously?  **Feedback: Yes** | We thank the reviewer for this encouraging comment. |
| 3. | Have the authors made all data underlying the findings in their manuscript fully available?  **Feedback: No** | We thank the reviewer for this comment. The reasons for not implementing this requirement are given in line 17 below. |
| 4. | Is the manuscript presented in an intelligible fashion and written in standard English?  PLOS ONE does not copyedit accepted manuscripts, so the language in submitted articles must be clear, correct, and unambiguous. Any typographical or grammatical errors should be corrected at revision, so please note any specific errors here.  **Feedback: Yes.** | We thank the reviewer for this encouraging comment. |
| 5. | The University of Botswana medical school was established in 2009 and the program evaluation was performed in 2016. Please consider deleting "new" from medical program in the title. | We thank the reviewer for this comment. We have removed ‘new’ from the title. |
| 6. | Have you performed a more recent program evaluation? If yes, did you use the same theoretical framework and evaluation model? What was the outcome of the recent evaluation compared to 2016? | The second review has not been carried out; however the approach to programme evaluation described in the manuscript will inform the next review. |
|  | **Background** | |
| 7. | The literature review provides an extensive and robust discussion of multiple theories and paradigms in program evaluation. However, this discussion is better suited as a scoping review on this topic. Please consider a succinct description of the general and contextual reasons for a program evaluation, the most frequently applied theoretical framework in program evaluation - including examples of institutions that have applied this framework successfully and why it was chosen. | - We thank the reviewer for these comments. - We have revised this section to make it more succinct and focused, also structuring it more clearly around the following: reasons for program evaluation (67-71), and frequently applied theoretical frameworks and models applied in program evaluation (89-121). In lines 127-131 reference is made to a scoping review of 44 studies reporting evaluations based on complexity theory, so this is not a new phenomenon; however none of those we located were evaluations of whole programmes. - The rationale for our using this model is given in lines 123-127. |
| 8. | You also provided a broad description of evaluation models. Please consider narrowing your discussion by summarizing these models and providing a rationale for the model you used for your program evaluation. |  |
| 9. | What are your SMART objectives for this evaluation process? | Thank you for the question. We had one broad objective for the study. To address the broad objective we then developed 90 specific objectives (phrased as questions), each of which we believe is SMART.   - *Broad objective:* To review the quality of the MBBS programme at the University of Botswana as it is currently implemented (plan, process and product) in relation to its initial aims and national and international standards. - *Specific objectives for this proposal*   For convenience the 90 objectives/ questions are appended in a Supporting Information document (S3 Text.Questions, data sources, instruments) |
|  | **The Approach** | |
| 10. | Lines 183 - 185 don't add any relevant information to this manuscript. | We thank the reviewer for this comment. We revised the statement to focus on the context of the programme, as follows – The University of Botswana Faculty of Medicine (UB FoM) offers a five-year, hybrid PBL undergraduate Bachelor of Medicine, Bachelor of Surgery (MBBS) curriculum (148-149) |
| 11. | Please provide the 90 evaluation questions that were derived from the evaluation standards as an appendix. | We thank the reviewer for this comment. They are provided in a Supporting Information document (S3 Text.Questions, data sources, instruments) |
| 12. | What criteria did you use to determine the appropriate data collection instrument for stakeholders? | We thank the reviewer for this comment. The nature of each question directed us to the data and then the data sources that we needed to achieve the answers to the question. The instruments used were selected according to (1) the data needed and the (2) sources from which the data could be obtained, so these two were our ‘criteria’ for selecting instruments (see S3 Text.Questions, data sources, instruments). In summary:   - Interview guides: for obtaining qualitative data from individuals - Focus group discussion guides: for obtaining qualitative data from groups of individuals whose interaction would be likely to enrich the data - Questionnaires: for obtaining quantitative as well as simple qualitative data from numbers of individuals, so that they could be summarised. - Observation guides: to guide the observation of activities in a structured way to provide information needed for particular study questions - Document study guide: see response below.   We followed the principle of triangulation throughout. An example is given in Table 4 – for that question seven data sources were used, each with appropriate instruments. |
| 13. | What is the purpose and content of the document study guide? | We thank the reviewer for this comment. By ‘document study guide’ we meant instruments which guided our reading of each document that had been identified as a data source for various research questions (e.g. when reading the MBBS Admissions Policy we had to look for information needed to answer 4 of the study’s research questions and the instrument guided us to do that). In the literature this is also called a ‘document analysis guide’. |
|  | **Validation** | |
| 14. | The introduction of Cilliers's ten characteristics of a complex system later in your manuscript poses another challenge in understanding the purpose of your research. Was your goal to validate the complexity theory in your institution's program evaluation? | - We thank the reviewer for this comment. We have now introduced our intention to use Cilliers in the ‘Background’ section (138-140). We also moved the analysis using the ten features of Cilliers from the end of the manuscript, to follow on the ‘six steps’. Our use of Cilliers is also mentioned in the Abstract. - Our goal was to use the features of a complex system (as set out by Cilliers), to validate our six-step approach: did our approach illuminate each of the ten features of this particular complex system? |
|  | **Financial disclosure** | |
| 15. | Please be informed that funding information should not appear in the Acknowledgments section or other areas of your manuscript. We will only publish funding information present in the Funding Statement section of the online submission form. Please remove any funding-related text from the manuscript.  Thank you for stating the following financial disclosure:  "No external funding was received to support this research. Planning workshops, data collection and data analysis expenses were fully funded by the Faculty of Medicine, University of Botswana."  At this time, please address the following queries:  a) Please clarify the sources of funding (financial or material support) for your study. List the grants or organizations that supported your study, including funding received from your institution.  b) State what role the funders took in the study. If the funders had no role in your study, please state:  c) If any authors received a salary from any of your funders, please state which authors and which funders.  d) If you did not receive any funding for this study, please state: “The authors received no specific funding for this work.”  Please include your amended statements within your cover letter; we will change the online submission form on your behalf. | Thank you for this comment. We have removed this information from the manuscript and will present it in the cover letter, as follows:  No external funding was received to support this research. Planning workshops, data collection and data analysis expenses were fully funded by the Faculty of Medicine, University of Botswana from its routine yearly budget. The funding authority in the Faculty of Medicine had no role in study design, data collection and analysis, decision to publish, or preparation of the manuscript. The researchers undertook the work as part of their routine academic duties and did not receive any additional remuneration. The authors (all of whom were researchers) also received no specific funding for this work. |
|  | **Data Related Comments** | |
| 16. | If applicable, we recommend that you deposit your laboratory protocols in protocols.io to enhance the reproducibility of your results. Protocols.io assigns your protocol its own identifier (DOI) so that it can be cited independently in the future. For instructions see: https://journals.plos.org/plosone/s/submission-guidelines#loc-laboratory-protocols. Additionally, PLOS ONE offers an option for publishing peer-reviewed Lab Protocol articles, which describe protocols hosted on protocols.io. Read more information on sharing protocols at <https://plos.org/protocols?utm_medium=editorial-email&utm_source=authorletters&utm_campaign=protocols> | Thank you for this comment. This is not relevant to our study which was not laboratory based. |
| 17. | Did you know that depositing data in a repository is associated with up to a 25% citation advantage (https://doi.org/10.1371/journal.pone.0230416)? If you’ve not already done so, consider depositing your raw data in a repository to ensure your work is read, appreciated and cited by the largest possible audience. You’ll also earn an Accessible Data icon on your published paper if you deposit your data in any participating repository (<https://plos.org/open-science/open-data/#accessible-data>).  In this instance, it seems there may be acceptable restrictions in place that prevent the public sharing of your minimal data. However, in line with our goal of ensuring long-term data availability to all interested researchers, PLOS’s Data Policy states that authors cannot be the sole named individuals responsible for ensuring data access.  Before we proceed with your manuscript, please also provide non-author contact information (phone/email/hyperlink) for a data access committee, ethics committee, or other institutional body to which data requests may be sent. | Thank you for this suggestion. In this case there are restrictions in place that prevent the public sharing of our minimal data. The consent forms we used asked study participants to agree to ‘provide the information requested, which will be incorporated (anonymously) *in the Review Report only’*. We are therefore not able to make these data available without restrictions.  We are following the alternative route you recommend, as follows:  Readers which wish to have access to the data supporting this research must please apply to: The Office for Research and Development, University of Botswana ([research.ethics@ub.ac.bw](mailto:research.ethics@ub.ac.bw)) |
|  | **Other Comments** |  |
| 18. | Your ethics statement should only appear in the Methods section of your manuscript. If your ethics statement is written in any section besides the Methods, please move it to the Methods section and delete it from any other section. | Thank you for this comment. We have moved the Ethics statement to the end of the Methods section (305-317) |
| 19. | Please include captions for your Supporting Information files at the end of your manuscript, and update any in-text citations to match accordingly. Please see our Supporting Information guidelines for more information: http://journals.plos.org/plosone/s/supporting-information | Thank you for this comment. We have done as you suggested for three files and have implemented your ‘Supporting Information’ guidelines |
|  | Please ensure that your manuscript meets PLOS ONE's style requirements, including those for file naming | Thank you for this comment. We have consulted the PLOS ONE ‘Style and Format’ guidelines and have adhered to them. |
| 20. | Reviewer #1: Please review the manuscript for typographical errors. | Thank you for this comment. We have checked the manuscript for typographical errors on several occasions and have addressed them. |
